# Potential role of extracellular vesicles in bacterial phagocytosis during *Escherichia coli* pneumonia in ex vivo perfused human lungs

**DOI:** 10.1186/s40635-026-00902-8

**Published:** 2026-04-29

**Authors:** Hongli He, Shinji Sugita, Qi Hao, Wonjung Hwang, Li Zhou, Yoshifumi Naito, Masaru Shimizu, Sandip Mukherjee, Michael A. Matthay, Jae-Woo Lee

**Affiliations:** 1 Department of Critical Care Medicine, Sichuan Provincial People’s Hospital, University of Electronic Science and Technology of China, Chengdu, Sichuan China; 2 Departments of Anesthesiology, Medicine, and Cardiovascular Research Institute, University of California, San Francisco, San Francisco, CA USA; 3 Present Address: Departments of Anesthesiology, University of California, Los Angeles, 757 Westwood Plaza Suite 3325, Los Angeles, CA 90095 USA

**Keywords:** Acute lung injury, Bacterial pneumonia, Extracellular vesicles, Ex vivo perfused human lung, MRP1 inhibitor, Reversan

## Abstract

**Background:**

Plasma extracellular vesicles (EV) play an inflammatory role in bacterial pneumonia. This study aimed to determine if plasma EVs could be manipulated to reduce lung injury. We hypothesized that inhibition of multidrug resistance-associated protein (MRP) 1 on EVs would increase total leukotriene (LT) B_4_ and lipoxin (LX) A_4_ levels and, when incubated with immune cells, would increase bacterial phagocytosis and decrease cytokines secreted by the cells.

**Results:**

Plasma EVs released from ex vivo perfused human lungs injured with *E.coli* bacteria (*E.coli* EV) or from control lungs (nEV) were incubated with LPS and Reversan, a MRP1 inhibitor, and the effect of EVs on bacterial phagocytosis and inflammation was studied using a macrophage cell line. In addition, the therapeutic effects of Reversan were studied in perfused human lungs injured with *E.coli* pneumonia. Levels of LTB_4_ and LXA_4_ were lower in *E.coli* EVs compared to nEVs. *E.coli* EVs were less capable than nEVs to stimulate bacterial phagocytosis by macrophages in vitro. Pretreatment of *E.coli* EVs with LPS and Reversan increased both total LTB_4_ and LXA_4_ levels, and, when incubated with Raw264.7 cells, bacterial phagocytosis was increased and TNFα levels secretion was decreased by the cells. Intravenous administration of Reversan to human lungs injured with *E.coli* bacterial pneumonia significantly restored alveolar fluid clearance rate and reduced bacterial levels in the injured alveolus at 6 h.

**Conclusion:**

Administration of a MRP1 inhibitor to plasma EVs increased its LTB_4_ content, which, when incubated with macrophages, further increased phagocytosis of bacteria by the cells.

**Supplementary Information:**

The online version contains supplementary material available at 10.1186/s40635-026-00902-8.

## Background

Acute respiratory distress syndrome (ARDS) due to pneumonia or acute lung injury (ALI) from sepsis remains a common cause of respiratory failure among critically ill patients [1]. Despite appropriate antibiotic use and supportive care, the mortality rate remains as high as 40% [2]. New therapeutic approaches are needed. Immune cells, such as macrophages or neutrophils play a critical role in host defense via the recognition, phagocytosis, and killing of pathogens [3]. The functional activity of these immune cells are often regulated by the environment through autocrine or paracrine mechanisms [3, 4]. Extracellular vesicles (EV), a heterogeneous population of membrane bound, anuclear microparticles released from the plasma membrane or from intracellular sources and comprised of exosomes, microvesicles and apoptotic bodies, are now recognized as important mediators of intercellular communication both in physiological and pathological states [5, 6]. Although the biological properties of EVs have been previously recognized, comparisons between studies in inflammatory states have been hindered by discrepancy in nomenclature and differences in isolation and characterization techniques for EVs. There is now a minimal standard for characterization of EVs which has resulted in convincing evidence that suggests that EV works through the transfer of EV content such as mRNAs, microRNAs, proteins, lipids and organelles to target cells [7–9]. For example, EVs released by GM-CSF stimulated monocytes induced target macrophage maturation/activation by transferring miR-223 and increased phagocytic activity by the cells [10]. Lee et al. reported that both epithelial cell and macrophage-derived microvesicles promoted macrophage migration and cytokine secretion through transfer of microRNA-17/221 during ALI [11, 12]. Although the role of EVs in propagating inflammation has been reported previously, the role of plasma EV in bacterial phagocytosis by host cells is largely unknown. Specifically, we now present data that suggest plasma EVs contain active compartments for lipid mediator regulation which can potentially enhance bacterial phagocytosis by target immune cells.

Lipids play an important role in regulating innate and adaptive immune response; multiple studies have demonstrated that leukotriene (LT) B_4_ enhanced host defense against pneumonia and sepsis via an increase in phagocytosis and release of antimicrobial agents by immune cells [13–15]. The biosynthesis of LTs occurs predominantly in leukocytes. In response to stimuli, arachidonic acid (AA) is liberated from membrane phospholipids, catalyzed by phospholipase A_2_. AA is then converted to LTA_4_ via the action of 5-lipoxygenase (5-LO). From LTA_4_ there are two competitive metabolic routes leading to the synthesis of LTB_4_ or LTC_4_ via the enzymes LTA_4_ hydrolase (LTA_4_H) or LTC_4_ synthase (LTC_4_S), respectively. LTC_4_ is transported across the plasma membrane by multidrug resistance-associated protein 1 (MRP1) and subsequently converted into LTD_4_ and LTE_4_ or cysteinyl LTs (CysLT) in the extracellular space. MRP1 is a member of the ATP-binding cassette transporter superfamily along with P-glycoprotein, also named ABCB1. MRP1 is a ~ 190 kDa membrane protein and functions as a transporter. LTC_4_ is a particularly high-affinity substrate of MRP1 [16, 17], and MRP1 is the major efflux pump for LTC_4_ [18]. In leukotriene-mediated inflammation, MRP1 plays a crucial role in LTC_4_ release [19, 20]. Surprisingly, mice lacking *Mrp1* are more resistant to bacterial pneumonia compared to wild type mice [20]. Owing to a lack of *Mrp1* expression, efflux of LTC_4_ is impaired and intracellular LTC_4_ is accumulated [20]. Accumulated LTC_4_ down-regulates LTC_4_S thus increasing the availability of LTA_4_ for LTA_4_H. LTA_4_ thus becomes more available for conversion to LTB_4_ leading to enhanced LTB_4_ and lipoxin (LX) A_4_ synthesis and release [21]. Recent studies have suggested that AA and its metabolites and biosynthetic enzymes were detectable in EVs during inflammation [22–24]. In the current study, we examined whether inhibition of MRP1 on plasma EVs could increase total LTB_4_ levels and whether this effect would increase bacterial phagocytosis by immune cells targeted by the EVs.

## Materials and methods

### Ex vivo lung perfusion

We have previously established the ex vivo perfused human lung as a model of severe bacterial pneumonia to enhance the clinical translation of any potential findings [25–28]. Research lungs (Northern California Transplant and One Legacy Donor Network) were perfused with DMEM plus 5% bovine serum albumin (volume = 1350 ml) with a cardiac output of 0.25 L/min and then ventilated (Harvard Apparatus, tidal volume 300 ml, respiratory rate = 10/min, FiO_2_ = 21%, and PEEP = 5 cmH_2_O)**.** Alveolar fluid clearance (AFC) rate was then measured in the control lung lobe by the change in protein concentration of a BAL fluid over 30 min: AFC (%/h) = 2(1-Ci/Cf) × 100 (Ci and Cf = protein concentration at 5 and 35 min, respectively). Then, 10^9^ CFU of *E.coli* bacteria K1 strain was instilled into the lower lobe, and fresh human blood (AB + , 100 ml) was added to the perfusate. After 6 h, the perfusate was collected to isolate EVs. In separate experiments, 20 mg Reversan, a selective inhibitor of MRP1 and P-glycoprotein, was administered intravenously 1 h after injury. Primary endpoint was the change in AFC rate in the injured lung lobe at 6 h.

### Isolation of extracellular vesicles

We and other investigators have established protocols to isolate and characterize both human and bacterial EVs for the study of lung injury [9, 28, 29]. Total perfusate was collected from control or bacterial injured lungs after 6 h of perfusion and was centrifuged at 2000 × g for 10 min, followed by 10,000 × g for 30 min to remove cellular debris and protein/lipid complexes. The final supernatant was then ultracentrifuged (Beckman Coulter Optima L-100XP 19 ultracentrifuge) at 100,000 × g at 4 °C for 1 h. The pellet was washed once with PBS prior to undergoing another ultracentrifugation. Then, the pellet containing EVs was resuspended with PBS (10 μl PBS per 1 ml perfusate) and stored at -80℃. During the isolation, the supernatant was not filtered nor was size exclusion chromatography or density gradient centrifugation for EV isolation performed due to the large volume utilized in the in vitro studies and concerns with the loss of recovery of EVs (> 50% in some studies) with these techniques [30]. To assess the level of contamination using ultracentrifugation, we measured BSA levels in the EV suspensions using an ELISA for BSA (GenScript); the perfusate contained 5% bovine serum albumin (BSA) or 50 mg/ml.

### Characterization of extracellular vesicles

Isolated nEVs and *E.coli* EVs from the perfusate of control and injured human lungs were characterized by morphology, size, protein and RNA content as recommended by the International Society of Extracellular Vesicles [8]. Morphology and structure of EVs were detected using transmission electron microscopy as previously described [31]. Size and number of EVs were analyzed using NanoSight NS300 (Malvern, Instruments, Malvern, U.K.). Parameters for NanoSight: 1) Camera Level: 14, Type: sCMOS, Laser Type: Green, 2) Detection Threshold: 7, 3) Sample Dilution Factor: 5 × 10^2^, 4) Number of Frames Analyzed: 749 and 5) Exclusion Criteria: None. The cellular sources of the EVs were previously characterized using flow cytometry [28]. The total protein levels were also assessed in EVs; nEVs or *E.coli* EVs were mixed with RIPA lysis buffer (Thermo Fisher Scientific, CA) supplemented with protease inhibitor cocktail (Sigma-Aldrich, St. Louis, MO, USA) to extract total protein. The concentration of total protein was detected using BCA protein assay kit (Thermo Fisher Scientific). The total RNA was extracted from nEVs or *E.coli* EVs using RNeasyMini Kit (QIAGEN Sciences, Germany), and the RNA content was quantified by NanoDrop ND1000 Spectrophotometer (Thermo Fisher Scientific). Critical inflammatory mRNA content of the EVs (i.e., TNFα, IL-1β and IL-6) were previously measured [28]. Comparison between nEVs and *E.coli* EVs was initially normalized based on the volume used to isolate the EVs after 6 h of perfusion with or without *E.coli* pneumonia. Because inflammation will change both the intra-vesicular content and increase the total EV number released [32], we believed that by normalizing by protein conc., mRNA/microRNA levels or particle number may dilute or underestimate the biological effects of these EVs seen in vitro or ex vivo. Consequently, the methodology used for Western blots can not lead to any conclusions in the expression of proteins in EVs released during *E.coli* pneumonia but only demonstrate the presence of these vesicle markers. However, to determine if the potency changes in the EV, in all direct comparisons between nEV and *E.coli* EV, we normalized the endpoint to per μg of protein. For all in vitro experiments involving Reversan, the control group received an equal volume of DMSO which was required to solubilize the MRP1 inhibitor. In addition, the DMSO used to solubilize Reversan increased LTB_4_ and LXA_4_ levels making direct comparisons between LPS [where no DMSO was administered] and Reversan + LPS pretreatment groups inaccurate (Fig. S1). Subsequently, all treated groups in these experiments were normalized to the control for comparisons.

### Statistics analysis

The data were presented as median with interquartile range (IQR). Comparisons between two groups were made using Mann–Whitney *U *test and between more than two groups using Kruskal–Wallis test with Dunn’s correction. A *P* value < 0.05 was considered statistically significant. Graphpad Prism 10 software was used for analysis. *P < 0.05, **P < 0.01, ***P < 0.001. Whether or not the samples were normally distributed was initially determined by D’Agostino and Pearson or Shapiro–Wilk normality test. However, for experiments with small sample size (N = 3–6), we chose nonparametric analyses to be more accurate. All comparisons between groups were unpaired and N represented the number of each experiment performed independently. All comparisons were considered primary analyses not exploratory given our previous work on MRP1 inhibition [33]. For CFU levels, the data was not log-transformed for statistical testing, only for presentation.

## Results

### Baseline demographic data for donor research lungs

Baseline demographic and clinical data for human research lungs used for experiments are listed in Table 1 and Table 2.

**Table 1:** Demographic and clinical data of donor lungs for isolation of plasma extracellular vesicles

	nEV (N = 3)	*E.coli* EV (N = 3)	*P* Value
Age (Years)	38 [35, 59]	36 [27, 43]	0.7
Female Sex, N (%)	3 (100%)	1 (33%)	
PaO_2_ (mmHg)	145 (111, 193)	110 (95, 129)	0.2
PaCO_2_ (mmHg)	43 (32, 43)	44 (43, 48)	0.2
PaO_2_/FiO_2_	278 (145, 483)	184 (119, 323)	0.7
PEEP (cmH_2_O)	10 (5, 10)	10 (5, 10)	> 0.9999
Cdyn (ml/cmH_2_O)	53 (36, 83)	40 (29, 46)	0.4
Murry LIS	1.3 (1.3, 2)	1.8 (1, 1.8)	> 0.9999
Ischemic Time (hours)	34 (29, 36)	40 (38, 47)	0.1

Data is presented as median with IQR for all variables. No significant differences were found in comparison between groups by Mann Whitney Test. nEV = EVs isolated from uninjured perfused human lungs. *E.coli* EV = EVs isolated from perfused human lungs injured with *E.coli* pneumonia

**Table 2:** Demographic and clinical data of donor lungs for lungs for *E.coli* Pneumonia ± Reversan Experiments

	*E.coli* Pneumonia (*N* = 5)	*E.coli* Pneumonia + Reversan (N = 5)	*P* Value
Age (Years)	48 (23, 54)	46 (28, 68)	0.69
Female Sex, N (%)	2 (40%)	3 (60%)	
PaO_2_ (mmHg)	155 (90, 498)	119 (96, 223)	0.84
PaCO_2_ (mmHg)	41 (34, 42)	33 (24, 39)	0.12
PaO_2_/FiO_2_	258 (171, 498)	293 (153, 339)	0.55
PEEP (cmH_2_O)	5 (5, 7.5)	5 (5, 9)	> 0.9999
Cdyn (ml/cmH_2_O)	35 (26, 42)	35 (29, 38)	> 0.9999
Murry LIS	1 (0.9, 2.1)	1.8 (0.7, 2)	0.90
Ischemic Time (hours)	29 (19, 38)	35 (31, 36)	0.55

Data is presented as median with IQR for all variables. No significant differences were found in comparison between groups by Mann Whitney Test

### Effect of Reversan on bacterial phagocytosis and lung protein permeability in human macrophages and lung microvascular endothelial cells

To determine the effects of a MRP1 inhibitor on potential target cells, we first exposed primary cultures of human macrophages to increasing levels of Reversan. Surprisingly, despite an increase in secreted LTB_4_ levels at lower doses and a decrease in secreted TNFα at higher doses, there was no significant effect on *E.coli* bacterial phagocytosis by the macrophages with Reversan. In addition, incubation of primary cultures of human lung microvascular endothelial cells injured with an inflammatory insult [i.e., cytomix, 50 ng/ml] as a surrogate for ARDS pulmonary edema fluid with Reversan further exacerbated protein permeability (Fig. S1). Given the lack of an effect of the inhibitor on target cells, we next focused on whether the MRP1 inhibitor changed plasma EVs. In our previous publication, we discovered that plasma EVs played a critical role in the pathogenesis of severe bacterial pneumonia [28].

### Characterization of extracellular vesicles

**Fig. 1:**
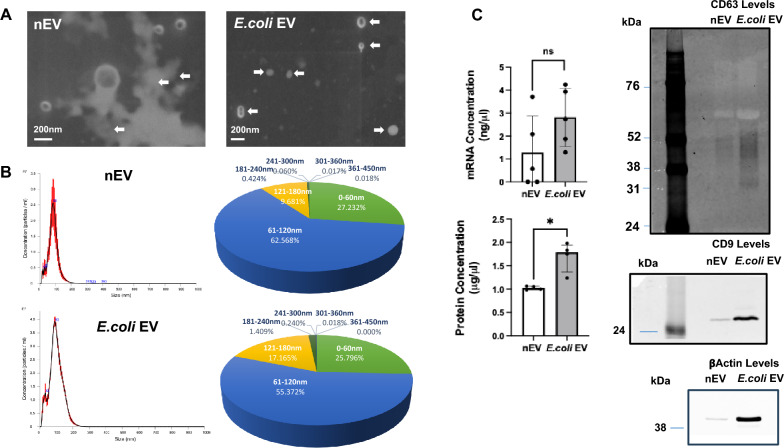
Characterization of Extracellular Vesicles Isolated from Ex Vivo Perfused Human Lungs with or without *Escherichia coli* Pneumonia. **A** By electron microscopy, EVs isolated from the plasma of both control perfused human lungs (nEV) or *E.coli* bacterial injured lungs (*E.coli* EV) were shown to be small membrane-bound vesicles (scale bar, 200 nm). Arrows highlight the different sizes of EVs. **B** By NanoSight analyses, 63% of the nEVs were 61–120 nm in size with a mean size of 83 nm while 55% of the *E.coli* EVs were 61–120 nm in size with a mean of 93 nm. **C** Compared with nEVs released from perfused human lungs without injury, *E.coli* EVs contained significantly higher levels of protein at 6 h. N = 5 for RNA, N = 4 for Protein levels. Data is expressed as median with interquartile range (IQR). Mann Whitney test was used for comparisons. When equal volumes of EVs were used, Western blot analyses demonstrated increased levels of canonical EV markers (CD9, CD63) and loading control (βActin) in *E.coli* EVs compared to nEVs which may be due in part to the higher levels of protein in *E.coli* EVs

EVs were isolated from the plasma of control [nEV] and *E.coli* bacteria [*E.coli* EV] injured lungs by ultracentrifugation only to increase EV yield. To assess the level of contamination, we found nEV and *E.coli* EVs in 1 ml of the perfusate following ultracentrifugation contained 0.33 ± 0.2 mg and 0.35 ± 0.3 mg of BSA or 0.7% and 0.7%, respectively, of the BSA originally in the perfusate. By electron microscopy, nEVs and *E.coli* EVs appeared as spheroids between 0 and 200 nm in size (Fig. 1A). The mean size of nEVs and *E.coli* EVs were 83 nm and 93 nm, respectively, as analyzed with NanoSight NS300 [Malvern Inc., UK] (Fig. 1B). Greater than 99% of nEVs and > 98% of *E.coli* EVs were < 180 nm in size. The concentration of nEVs was 2.6 × 10^11^ particles/ml compared to 3.9 × 10^11^ particles/ml for *E.coli* EVs (N = 3, *P* = 0.42). There were no significant differences between the groups in terms of size distribution and total RNA level. However, total protein levels were significantly higher in *E.coli* EVs than in nEVs (Fig. 1C). To demonstrate the presence of canonical EV markers, Western blots for CD9, CD63 and βActin were performed using equal volume of each EV preparation (Fig. 1C). Similar to the total protein conc. and total particle number/ml, there was an obvious increase in CD9, CD63 and βActin levels in *E.coli* EV compared to nEV at 6 h. Currently, we cannot conclude any direct effects of bacterial pneumonia to changes in the expression of these proteins, only on their presence in EVs. Consequently, in all direct comparisons between nEV and *E.coli* EV, we normalized the endpoint to per μg of protein.

### Effect of LPS on leukotriene levels in *E. coli* EVs

**Fig. 2:**
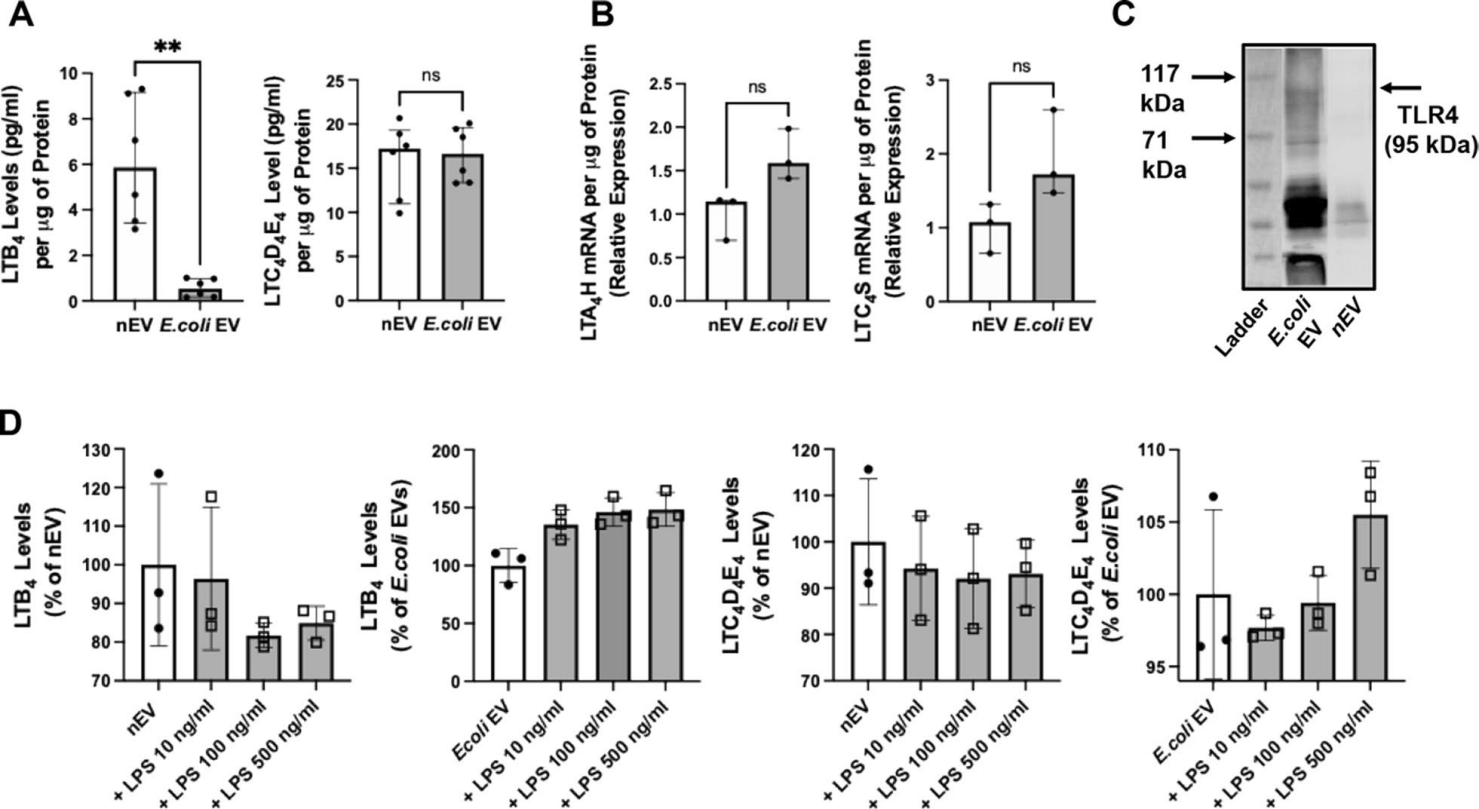
Expression of Leukotrienes in Extracellular Vesicles. **A** By ELISA, the total expression of LTB_4_ in *E.coli* EVs was lower than in nEVs. **B** Gene expression of LTC_4_S and LTA_4_H were both numerically higher in *E.coli* EVs by Real-time PCR. **C** *E.coli* EVs expressed significant levels of TLR4 by Western Blot analysis. **D** LTB_4_ EV levels did not change with LPS stimulation in nEVs. However, there was a dose response increase to LPS in LTB_4_ levels in *E.coli* EVs. There were no significant changes in CysLTs in both nEVs and *E.coli* EVs. Data is expressed as median with IQR, N = 6 for (**A** for leukotriene levels), N = 3 for (**B)** and (**D)**. Mann Whitney test were used for (**A)** and (**B)**, Dunn’s test following Kruskal Wallis analysis was performed for (**D)**

Compared with nEVs, LTB_4_ levels were significantly lower in *E.coli* EVs at baseline (Fig. 2A). MRNA levels of the enzymes LTC_4_S and LTA_4_H, which are responsible for the synthesis of CysLTs and LTB_4_, respectively in immune cells, were numerically higher in *E.coli* EVs compared to nEVs (Fig. 2B). Although no conclusion can be made due to different loading amounts, TLR4 protein levels in *E.coli* EVs were more prominent than in nEVs by Western blot (Fig. 2C). Although not statistically significant, exposure of *E.coli* EVs to LPS numerically increased total LTB_4_ levels in a dose dependent manner by up to 43%, while LPS had no effect on nEVs. There were no differences in CysLTs levels in both nEVs and *E.coli* EVs after LPS stimulation (Fig. 2D). For ELISAs, we currently cannot distinguish extracellular/surface-associated LTs from intravesicular sources due to the methodology used to lysis the EVs.

### Effect of extracellular vesicles on phagocytosis of bacteria by Raw264.7 macrophages

**Fig. 3:**
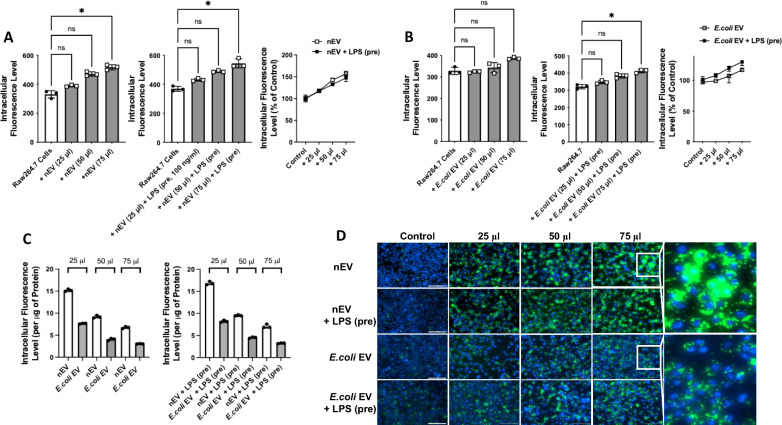
Effect of Extracellular Vesicles on the Phagocytosis *E.coli* Bacteria by Raw264.7 Macrophages. **A** When exposed to nEVs, the phagocytosis of GFP labeled *E.coli* bacteria by Raw264.7 cells increased significantly in a dose dependent manner. LPS pretreatment of nEVs prior to exposure to Raw264.7 cells had no additional effects on *E.coli* bacteria phagocytosis. **B** When exposed to *E.coli* EVs, the phagocytosis of GFP labeled *E.coli* bacteria by Raw264.7 cells increased in a dose dependent manner. LPS pretreatment of *E.coli* EV prior to exposure to Raw264.7 cells further increased the phagocytosis of bacteria. **C** The effect of nEVs on phagocytosis of *E.coli* bacteria by Raw264.7 cells was numerically greater when compared to the effect of *E.coli* EVs at all doses. After LPS pretreatment, the effect of *E.coli* EVs on phagocytosis increased, however, per μg of EV protein, the difference between LPS pretreated nEVs and LPS pretreated *E.coli* EVs on macrophage phagocytosis was unchanged. **D** Representative fluorescence images of phagocytosis of GFP labeled *E.coli* bacteria by Raw264.7 cells of N = 3 independent experiments. For each assessment, a defined number of fields was selected and quantified in an automated fashion. Scale bars = 100 μm. Data were expressed as median with IQR. N = 3 for all groups. Dunn’s test following Kruskal Wallis analysis was performed for multiple comparisons. (pre) = pretreatment of the EVs

The antimicrobial effect of EVs on the phagocytosis of GFP labeled *E.coli* bacteria by Raw264.7 cells were assessed by measuring intracellular fluorescence. Exposure of Raw264.7 cells to nEVs significantly increased the phagocytosis of *E.coli* bacteria in a dose-dependent manner (Fig. 3A). Pretreatment of nEVs with LPS prior to exposure to Raw264.7 cells did not further increase the phagocytosis of bacteria. Exposure of Raw264.7 cells to *E.coli* EVs enhanced the phagocytosis of bacteria by Raw 264.7 in a dose dependent manner (Fig. 3B). Pretreatment of *E.coli* EVs with LPS further increased the phagocytosis of bacteria by Raw264.7 cells. However, when compared with nEVs per μg of EV protein, the effect *E.coli* EVs in phagocytosis was lower at all doses with or without LPS pretreatment (Fig. 3C). For all subsequent experiments, we choose the EV treatment dose of 75 μl.

### MRP1 inhibitor increased total LTB_4_ levels in ***E.coli*** EVs and enhanced the effect of EV on bacterial phagocytosis by Raw264.7 cells

**Fig. 4:**
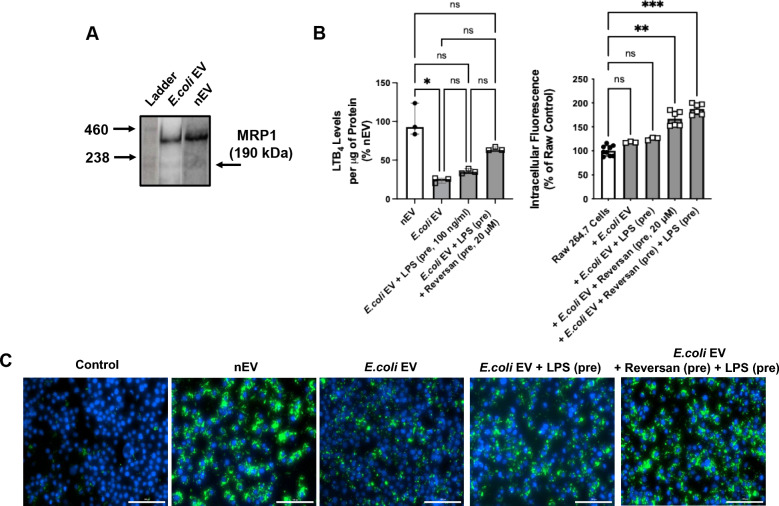
Effect of MRP1 Inhibitor on Total LTB_4_ Levels in *E.coli* EV and the Effect of Pretreated EV on the Phagocytosis of Bacteria by Raw264.7 cells. **A** By Western blot analyses, MRP1 protein was expressed in both nEVs and *E.coli* EVs. **B** Pretreatment of *E.coli* EVs with LPS and Reversan, a MRP1 inhibitor, increased LTB_4_ levels in *E.coli* EVs. Pretreatment of *E.coli* EVs with LPS with Reversan also significantly increased the phagocytic capacity of Raw264.7 cells. (**C)** Representative fluorescent images of phagocytosis of GFP labeled *E.coli* bacteria by Raw264.7 cells treated with EVs with LPS and a MRP1 inhibitor of N = 3 independent experiments. For each assessment, a defined number of fields was selected and quantified in an automated fashion. Scale bars = 100 μm. Data were expressed as median with IQR. N = 3 for all treatment groups. Dunn’s test following Kruskal Wallis analysis was performed. (pre) = pretreatment of the EVs. See Method Section for the rationale to normalize to DMSO control

Preliminary experiments were performed to determine the optimal dose of the MRP1 inhibitor, Reversan, in increasing total LTB_4_ levels in *E.coli* EVs (Fig. S2). For all subsequent studies, Reversan at a dose of 20 μM was used. Pretreatment of *E.coli* EVs with LPS and Reversan restored LTB_4_ levels to levels closer to nEVs (Fig. 4B). We next assessed the effect of MRP1 inhibitor pretreatment of *E.coli* EVs on bacterial clearance by Raw264.7 cells. Pretreatment of *E.coli* EVs with Reversan and LPS significantly increased the phagocytosis of bacteria by Raw264.7 cells (Fig. 4B). As controls, there were no effects of equivalent doses of LPS, DMSO, and Reversan alone used for pretreatment of EVs on bacteria phagocytosis by Raw264.7 cells (data not shown).

### Preincubation of *E. coli* EVs with MRP1 inhibitor decreased the secretion of TNFα by LPS injured Raw264.7 cells

**Fig. 5:**
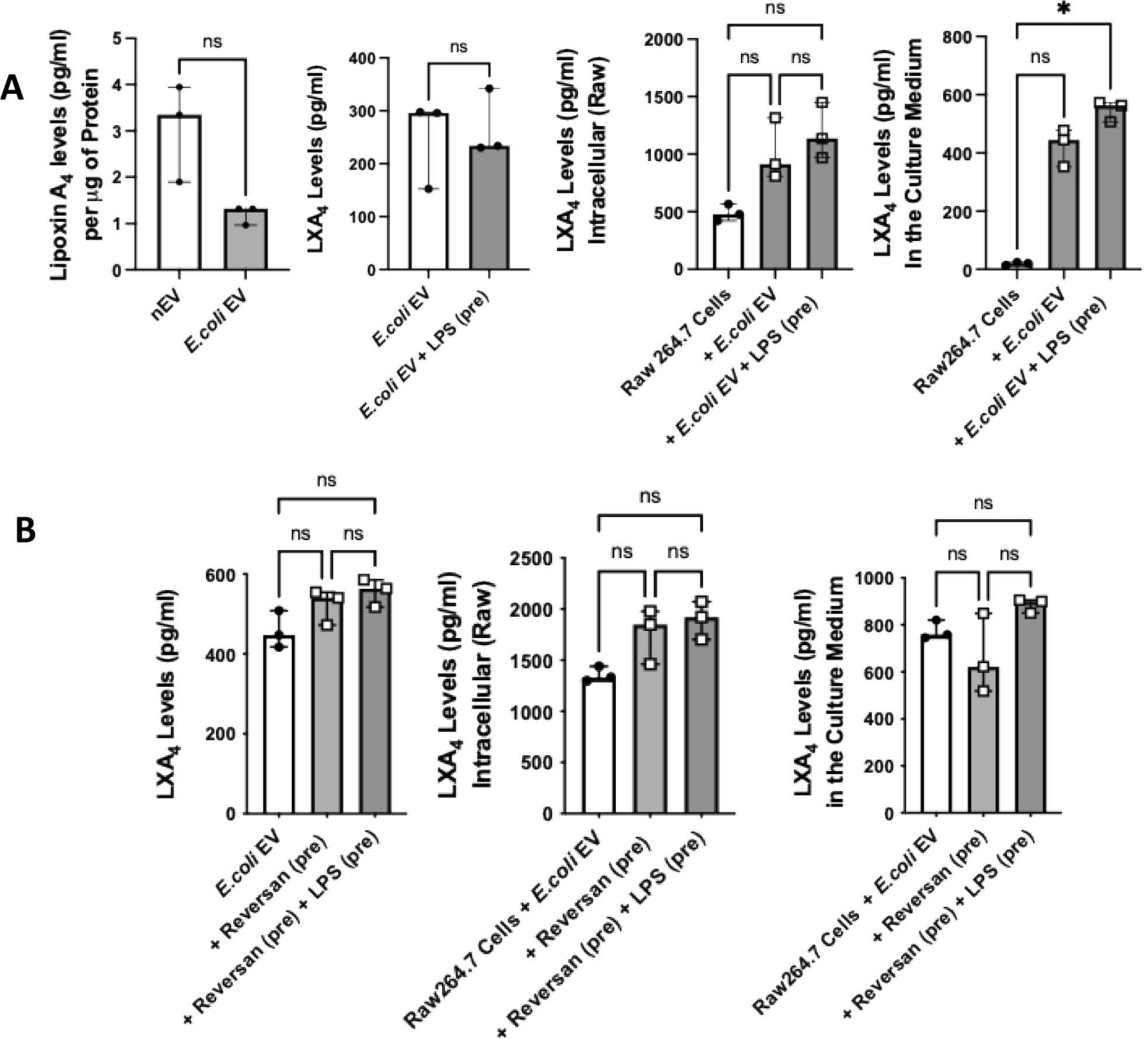
Effect of LPS with MRP1 Inhibition on *E.coli* EV Total LipoxinA_4_ Levels and on Raw264.7 Intracellular and Secreted LipoxinA_4_ Levels. **A** Total levels of LXA_4_ were numerically lower in *E.coli* EVs compared to nEVs. Treatment of *E.coli* EVs with LPS had no effect on LXA_4_ levels. However, co-incubation of Raw264.7 cells with *E.coli* EVs pretreated with LPS significantly increased Raw264.7 secreted LXA_4_ levels. **B** Pretreatment of *E.coli* EVs with Reversan and LPS numerically increased LXA_4_ level by 26% over EV alone. Exposure of Raw264.7 cells with *E.coli* EVs pretreated with Reversan and LPS increased Raw264.7 intracellular and secreted LXA_4_ levels by 44% and 18% respectively. Data were expressed as median with IQR, N = 3 for treatment groups. Mann Whitney test or Dunn’s test following Kruskal Wallis analysis was performed. (pre) = pretreatment of the EVs. See Method Section for the rationale to normalize to DMSO control

At baseline, nEVs contained numerically higher levels of total LXA_4_ than *E.coli* EVs. Although there was no effect of LPS on total LXA_4_ levels in *E.coli* EVs, administration of LPS pretreated *E.coli* EVs to Raw264.7 cells increased total secreted levels of LXA_4_ (Fig. 5A). Incubation of Raw264.7 cells with LPS and Reversan pretreated *E.coli* EVs further increased intracellular and secreted LXA_4_ levels by an additional 44% and 18%, respectively (Fig. 5B).

**Fig. 6:**
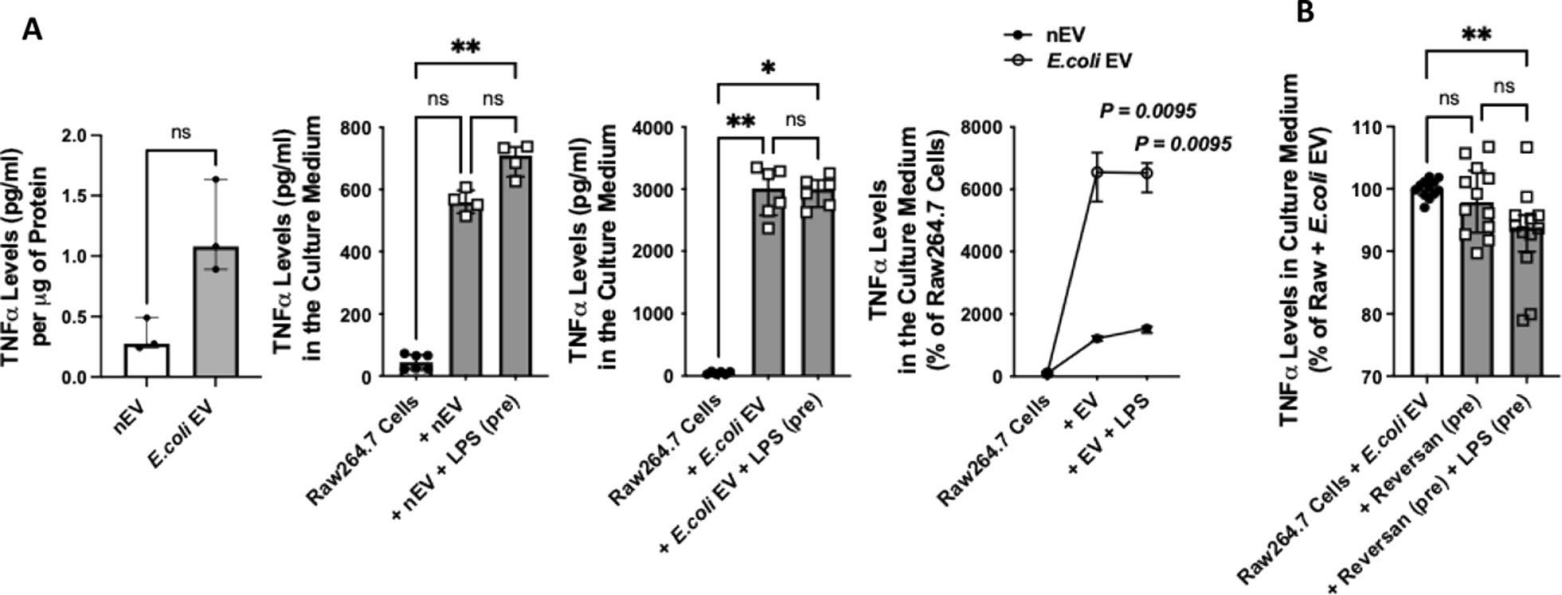
Effect of MRP1 Inhibition on the Inflammatory Properties of *E.coli* EV on Raw264.7 Cells. **A** At baseline, total TNFα levels were numerically higher in *E.coli* EVs compared to nEVs. However, incubation of Raw264.7 cells with either nEVs or *E.coli* EVs with or without LPS pretreatment significantly increased the secretion of TNFα by Raw264.7 cells. Compared to nEVs, the effect of *E.coli* EVs on the secretion of TNFα by Raw264.7 cells were 5X higher at baseline and 4X higher with LPS pretreatment. **B** Pretreatment of *E.coli* EVs with LPS and Reversan significantly reduced the secretion of TNFα by Raw264.7 cells. Data were expressed as median with IQR. N = 3—6 for (**A)** and N = 12 for (**B)**. Mann Whitney test or Dunn’s test following Kruskal Wallis analysis was performed for statistical analyses. (pre) = pretreatment of the EVs

At baseline, total TNFα levels were higher in *E.coli* EVs compared to nEVs. When incubated with Raw264.7 cells, both nEVs and *E.coli* EVs caused significant secretion of TNFα by the cells. However, the effect of *E.coli* EVs were > 5X higher (Fig. 6A). Incubation of Raw264.7 cells with LPS and Reversan pretreated *E.coli* EVs decreased TNFα levels by 7% (Fig. 6B). Although statistically significant, given the variability in TNFα responses across experiments, the decrease in inflammation was modest.

### Effect of Reversan administration in ex vivo perfused human lungs injured with *E.coli* pneumonia

**Fig. 7:**
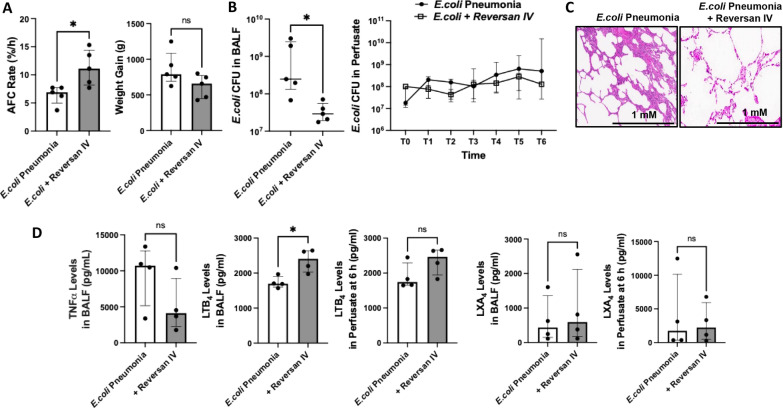
Therapeutic Effects of Reversan Administration in the Ex Vivo Perfused Human Lungs Injured with Severe *E.coli* Pneumonia. **A** Intravenous instillation of Reversan 20 mg 1 h following initiation of *E.coli* pneumonia significantly increased AFC rate and numerically decreased lung weight gain at 6 h. **B** More importantly, administration of Reversan significantly decreased the bacterial CFU levels in the injured alveolus. **C** Representative histology of human lungs injured with *E.coli* bacteria with and without treatment with IV Reversan. However, no lung injury score was performed on the histology given the patchy nature of the pneumonia which is a limitation to the model. Magnification 4X. Scale bars = 1 mM. **D** Administration of Reversan numerically decreased TNFα levels in the injured alveolus by 62%. The decrease in inflammation and bacterial CFU counts was also associated with a significant increase in LTB_4_ in the injured alveolus with Reversan therapy by 42%. Data is expressed as median with IQR, N = 4 − 5. Mann Whitney test were used for comparisons. For lungs injured with *E.coli* pneumonia, an equal volume of DMSO used to solubilize Reversan was administered IV at 1 h following injury as controls

A schematic of the protocol for the ex vivo perfused human lung experiments is shown in (Fig. S3). Intravenous administration of Reversan 20 mg as therapy, giving a plasma level of 20–30 μM, significantly restored AFC rate in perfused human lungs injured with *E.coli* bacterial pneumonia [median with IQR: 6.9 (4.9, 7.7) vs 11.1 (8.2, 14.4), *P* = 0.0238] at 6 h which was associated with a decrease in lung weight gain (Fig. 7A). More importantly, administration of Reversan significantly decreased bacterial CFU levels in the injured alveolus [median with IQR: 2.5 × 10^8^ CFU [1.3 × 10^8^, 2.5 × 10^9^] vs 2.9 × 10^7^ [1.9 × 10^7^, 5.5 × 10^7^]], *P* = 0.0159 (Fig. 7B), which was associated with increased LTB_4_ levels in the injured alveolus and perfusate by 42% and 41%, respectively. Although there were no changes in LXA_4_ levels in the injured alveolus, administration of Reversan numerically decreased TNFα levels by 62% (Fig. 7D). Incubation of human macrophages with LPS and Reversan pretreated *E. coli* EVs significantly increased bacterial phagocytosis by 50% (Fig. S4).

### Effect of zileuton on total LTB_4_ and LXA_4_ levels in ***E.coli*** EVs

**Fig. 8:**
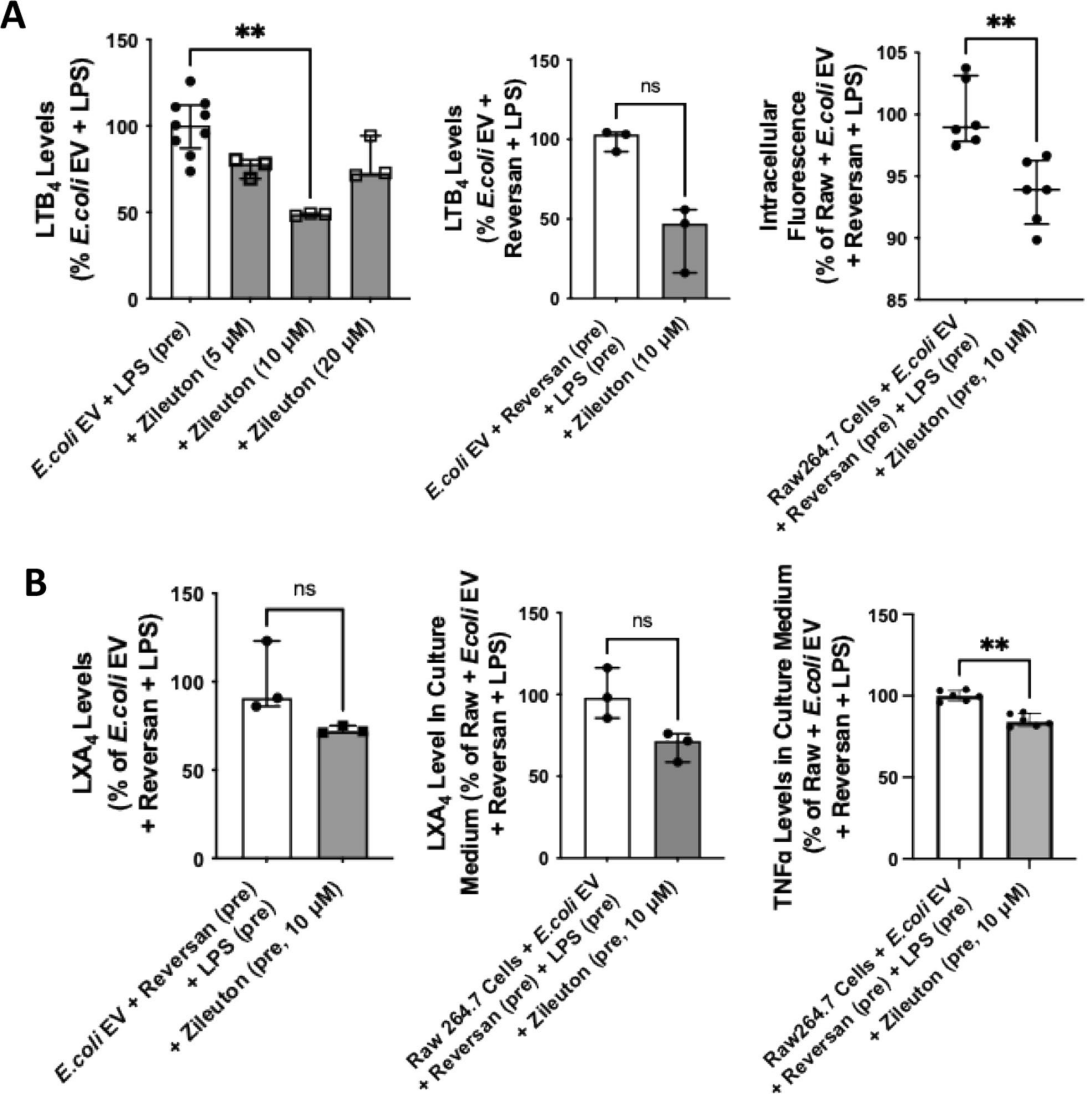
Effect of 5-Lipoxygenase Inhibitor on the Synthesis and Effect of LTB_4_ in *E.coli* EVs. **A** Incubation of *E.coli* EVs with Zileuton, a 5-LO Inhibitor, decreased the total levels of LTB_4_ in a dose dependent manner; 5-lipoxygenase is the initial enzyme in the biosynthesis of leukotrienes from arachidonic acid. The effect of Zileuton on LTB_4_ levels in EVs was apparent whether or not *E.coli* EVs were pretreated with LPS and Reversan. Zileuton also inhibited the increase in bacteria phagocytosis by Raw264.7 cells, demonstrating the importance of increased LTB_4_ in EVs in bacterial clearance by the macrophage cell line. **B** Incubation of pretreated *E.coli* EVs with Zileuton also numerically decreased total LXA_4_ levels in the EVs and the secretion of LXA_4_ by Raw264.7 cells. However, treatment with Zileuton decreased TNFα secretion by these Raw264.7 cells when incubated with the *E.coli* EVs. This effect is probably the result of inhibition of cysteinyl LTs or LTC_4_D_4_E_4_ synthesis by Zileuton. Data were expressed as median with IQR. N = 3 − 6 per treatment groups. Mann Whitney test and Dunn’s test following Kruskal Wallis analysis was performed for comparisons. (pre) = pretreatment of the EVs

Incubation of LPS and Reversan pretreated *E.coli* EVs with Zileuton, a 5-LO inhibitor, decreased total LTB_4_ levels. When incubated with Raw264.7 cells, the addition of Zileuton to *E.coli* EVs decreased bacterial phagocytosis (Fig. 8A). Incubation of LPS and Reversan pretreated *E.coli* EVs with Zileuton also numerically decreased LXA_4_ levels. When incubated with Raw264.7 cells, the addition of Zileuton decreased LXA_4_ levels in the culture medium which was associated with a decrease in TNFα levels as well (Fig. 8B).

## Discussion

The main findings of this study can be summarized as follows: 1) Levels of LTB_4_ in the perfusate EVs were significantly different depending on whether the lungs were injured with pneumonia; LTB_4_ level was lower in *E.coli* EVs compared to nEVs (Fig. 2); 2) *E.coli* EVs expressed both TLR4 and MRP1 by Western blot analyses. Incubation with LPS and MRP1 inhibitor increased LTB_4_ levels in *E.coli* EVs (Figs. 2 and 4); 3) Incubation with LPS and a MRP1 inhibitor increased the effect of *E.coli* EVs on phagocytosis by Raw264.7 cells and decreased inflammation (Figs. 3, 4, 5, 6); 4) Treatment of *E.coli* EVs with Zileuton, a 5-LO inhibitor, blocked the effect of the MRP1 inhibitor (Fig. 8); 5) In ex vivo perfused human lung injured with *E.coli* bacterial pneumonia, intravenous administration of Reversan restored AFC rate and reduced inflammation and total bacteria counts in the injured alveolus (Fig. 7). Human macrophages exposed to plasma EVs isolated from *E.coli* bacteria injured lungs pretreated with LPS and Reversan had increased phagocytosis activity (Fig. S4). Taken together, our study revealed for the first time that MRP1 inhibitors increased LTB_4_ levels in *E.coli* EVs and enhanced the ability of *E.coli* EVs to promote bacterial phagocytosis and possible clearance by immune cells (Fig. S5).

EVs are important messengers for intercellular communication. Although there are significant overlap, exosomes are released from intracellular compartments with a diameter of 20–100 nm while microvesicles are generated from outward budding off the plasma membrane with a diameter of 100–1000 nm in response to diverse physiological, pathophysiological or external stimulus [28, 34]. In our previous publication, we found that 60% of *E.coli* EVs expressed CD9, a marker of exosomes, and 28% expressed CD44, a marker of microvesicles [28] and originated predominantly from endothelial cells and platelets, and, to lesser extent, from epithelial cells, lymphocytes, red blood cells, monocytes, or macrophages [28, 35].

EVs work as messengers through the transfer of intra-vesicular content such as mRNA, microRNA, proteins, and organelles. Most previous studies on the biological effects of EVs have focused on proteins and nucleic acids. However, EVs are also enriched with phospholipids. With stimulation, AA can be released from the membrane and be metabolized into prostaglandins or leukotrienes by the cyclooxygenase and lipoxygenase pathways respectively, which can be transported through EVs [17, 22, 24]. With lung injury, we found LTB_4_ level was decreased in *E.coli* EVs compared with nEVs. Interestingly, mRNAs for both LTC_4_S and LTA_4_H, which are involved in LTC_4_ and LTB_4_ synthesis, were numerically higher in *E.coli* EVs compared to nEVs (Fig. 2). More importantly, at baseline, nEVs had more phagocytosis effect on target immune cells than *E.coli* EVs.

LPS is a critical virulence factor released by Gram negative bacteria which can affect both immune cells and possibly its’ released EVs. We found that LPS stimulation significantly increased LTB_4_ expression in *E.coli* EVs in a dose dependent manner but had no effect on nEVs which may be due to changes in TLR4 expression on immune cells and its’ released EVs following bacterial pneumonia [36]; TLR4 could only be detected in *E.coli* EVs although the amount of protein used for the Western blot was higher in *E.coli* EVs compared to nEVs (Fig. 2). More importantly, the increase in LTB_4_ levels in *E.coli* EVs indicated that LPS can stimulate EVs just like the parent immune cells [37].

Multiple studies have confirmed that plasma EVs participated in the pathogenesis of ALI [7, 28]; specifically, EVs increased macrophage and neutrophil influx and vascular permeability in the injured alveolus, leading to increased inflammation [38]. In the current study, the effect of EVs from control and injured lungs on the phagocytosis of bacteria by Raw264.7 macrophage was measured. Phagocytosis of fluorescent labelled *E.coli* bacteria by macrophages in vitro increased in a dose dependent manner after incubation with nEVs. Surprisingly, the effect of unstimulated *E.coli* EVs on phagocytosis was significantly lower. However, following pretreatment with LPS, *E.coli* EVs increased the bacterial clearance by macrophages. The change in phagocytotic activity was in accordance with the LTB_4_ level in the *E.coli* EVs. Previous studies demonstrated the antimicrobial effects of LTB_4_ in pneumonia and sepsis [14, 15]. Our study provided new insight that EVs may play an important role in maintaining the antimicrobial function of macrophages or other immune cells in homeostasis and during lung injury (Fig. 3). This function of plasma EVs, to migrate easily into the injured alveolus due to its small size and enhance the antimicrobial activity of target cells, may be an early biological response to ALI prior to the migration of professional phagocytes.

The increase in LTB_4_ levels in *E.coli* EVs following incubation with LPS and the presence of the enzymes required for LT synthesis in EVs provided us important clues that LT synthesis in EVs could be manipulated. A previous study showed that inhibition of MRP1 could increase LTB_4_ expression in macrophages in vitro and suppressed indices of ALI in mice with bacterial pneumonia [33]. By Western blot analyses, MRP1 receptor was expressed in *E.coli* EVs. Thus, we pretreated *E.coli* EVs with a MRP1 inhibitor, Reversan. Surprisingly, the concentration of LTB_4_ in *E.coli* EVs increased further (Fig. 4). The enzyme, 5-LO, plays a critical role in LTB_4_ synthesis. Treatment of *E.coli* EVs with a 5-LO inhibitor, Zileuton, prior to incubation with LPS and MRP1 inhibitors reduced LTB_4_ production. These results demonstrated that LTB_4_ synthesis may be upregulated by MRP1 inhibitors (Fig. 8). We next determined the function of *E.coli* EVs pretreated with LPS and MRP1 inhibitors on bacterial clearance by Raw264.7 cells. Pretreatment of *E.coli* EVs with Reversan significantly increased the phagocytosis of bacteria by the macrophage cell line, which was associated with increased LTB_4_ level in the EVs. This effect was inhibited with Zileuton (Fig. 8).

Immune cells play a critical role in propagating inflammation during ALI. In the current study, we found that incubation of Raw264.7 cells with *E.coli* EVs increased the secretion of TNFα by the macrophages. However, pretreatment of *E.coli* EVs with Reversan partially suppressed TNFα secretion in the culture medium (Fig. 6). One possible explanation was the MRP1 inhibition also produced a soluble factor with immunomodulatory properties. LXA_4_, an eicosanoid product generated from AA, is a member of the specialized pro-resolving mediator family of polyunsaturated fatty acid metabolites which act to resolve inflammatory responses [39, 40]. We found that LPS and MRP1 inhibition led to increased total LXA_4_ levels in *E.coli* EVs. When incubated with Raw264.7 cells, these EVs increased both intracellular and secreted LXA_4_ levels which could be inhibited with Zileuton (Figs. 5 and 8). This suggested that pretreatment of *E.coli* EVs with LPS and MRP1 inhibitor not only augmented phagocytosis of bacteria by macrophages but also ameliorated inflammation.

To increase clinical significance, we tested the therapeutic effects of MRP1 inhibitor in ex vivo perfused human lungs injured with *E.coli* bacterial pneumonia. Administration of intravenous Reversan restored AFC rate and numerically reduced total lung weight gain suggesting a reduction in pulmonary edema. The improvement in fluid clearance was associated with decreased inflammation and bacterial load in the injured alveolus at 6 h. Administration of Reversan also increased LTB_4_ levels in the injured alveolus and perfusate, although LXA_4_ levels were unchanged (Fig. 7). When administered to human macrophages in vitro, Reversan pretreated *E.coli* EVs also increased the phagocytosis activity of the macrophages (Fig. S4).

There are some limitations to the current study: 1) Sample size is inherently constrained due to the lack of availability of research lungs; consequently, some of the mechanistic analyses (i.e., N = 3) are supportive but not definitive. Larger sample sizes are needed to fully evaluate some inflammatory endpoints; 2) We were unable to demonstrate that incubation with Reversan alone increased bacterial phagocytosis by human macrophages (Fig. S1). This unexpected finding may be due to off target effects of the MRP1 inhibitor, an area which we are currently studying; 3) In the measurements of LTB_4_ and LXA_4_ by ELISA, we were unable to distinguish extracellular/surface-associated levels of these LTs from intravesicular content due to the methodology used; 4) We only studied EVs isolated from the perfusate. The cellular origins and the biological effects of EVs may be different if the EVs were collected from another source such as from the injured human alveolus; 5) Despite the focus on LTB_4_, LXA_4_ can also improve phagocytic activity of macrophages via NFE2L2 or through reorganization of the cytoskeleton [41, 42]; 6) Lack of other immune organs such as the spleen or liver which may participate in injury and/or repair or the elimination of EVs; 7) Lack of study on the contributions of EVs released from bacteria itself on ALI [43]; 8) And methodological constraints, such as normalization strategies for EV and in vitro assays such as the fluorescence-based phagocytosis assays (e.g. internalization vs. attachment/adhesion). For example, in comparisons between EVs from control and injured lungs by Western blot, no conclusions can be made on the effect of *E.coli* bacteria on vesicle markers such as TLR4 due to different loading amounts.

## Conclusions

In addition to both the innate and adaptive immune system, host defense again bacterial pneumonia involves other mechanisms including anatomical barriers, mucociliary clearance, alveolar lining fluid with surfactant proteins and nonspecific antimicrobial peptides. Even recent evidence suggests that other organs such as the gut microbiota may be involved [44]. In the literature, the role of plasma EVs in the host response has evolved from direct bacteriostatic properties to, more recently, the delivery of mRNA, miRNA, and protein to immune cells [33, 45]. In the current study, we found that plasma EVs isolated from human lungs injured with *E.coli* bacterial pneumonia were less capable than nEVs isolated from uninjured human lungs to stimulate bacterial phagocytosis by macrophages. However, pretreatment of *E.coli* EVs with LPS and MRP1 inhibitors increased LTB_4_ levels and restored the ability of EVs to stimulate bacterial phagocytosis by macrophages compared to nEVs. In addition, pretreatment of *E.coli* EVs with LPS and MRP1 inhibitors increased target macrophages’ LXA_4_ intracellular and secreted levels which resulted in reduced inflammation (Fig. S5). In ex vivo perfused human lungs injured with *E.coli* bacterial pneumonia, intravenous administration of Reversan restored AFC rate and decreased inflammation and bacterial load in the injured alveolus. Consistent with the in vitro studies, administration of Reversan increased LTB_4_ levels in the injured alveolus and perfusate. Targeting plasma EVs may be a therapeutic approach for enhancing the host response to ARDS.

## Supplementary Information

Additional file1 (PPTX 1664 KB)

Additional file2 (DOCX 22 KB)

## Data Availability

All data relevant to the study are included in the article or uploaded as supplementary information.
