# Supplementary material for: Potential role of extracellular vesicles in bacterial phagocytosis during Escherichia coli pneumonia in ex vivo perfused human lungs
Source: Intensive Care Med Exp. 2026 Apr 29;14:56. doi: 10.1186/s40635-026-00902-8 (PMC13129058; doi:10.1186/s40635-026-00902-8)
Supplement: Supplementary file 1 — Additional file1 (PPTX 1664 KB) [file 40635_2026_902_MOESM1_ESM.pptx]

## Slide 1
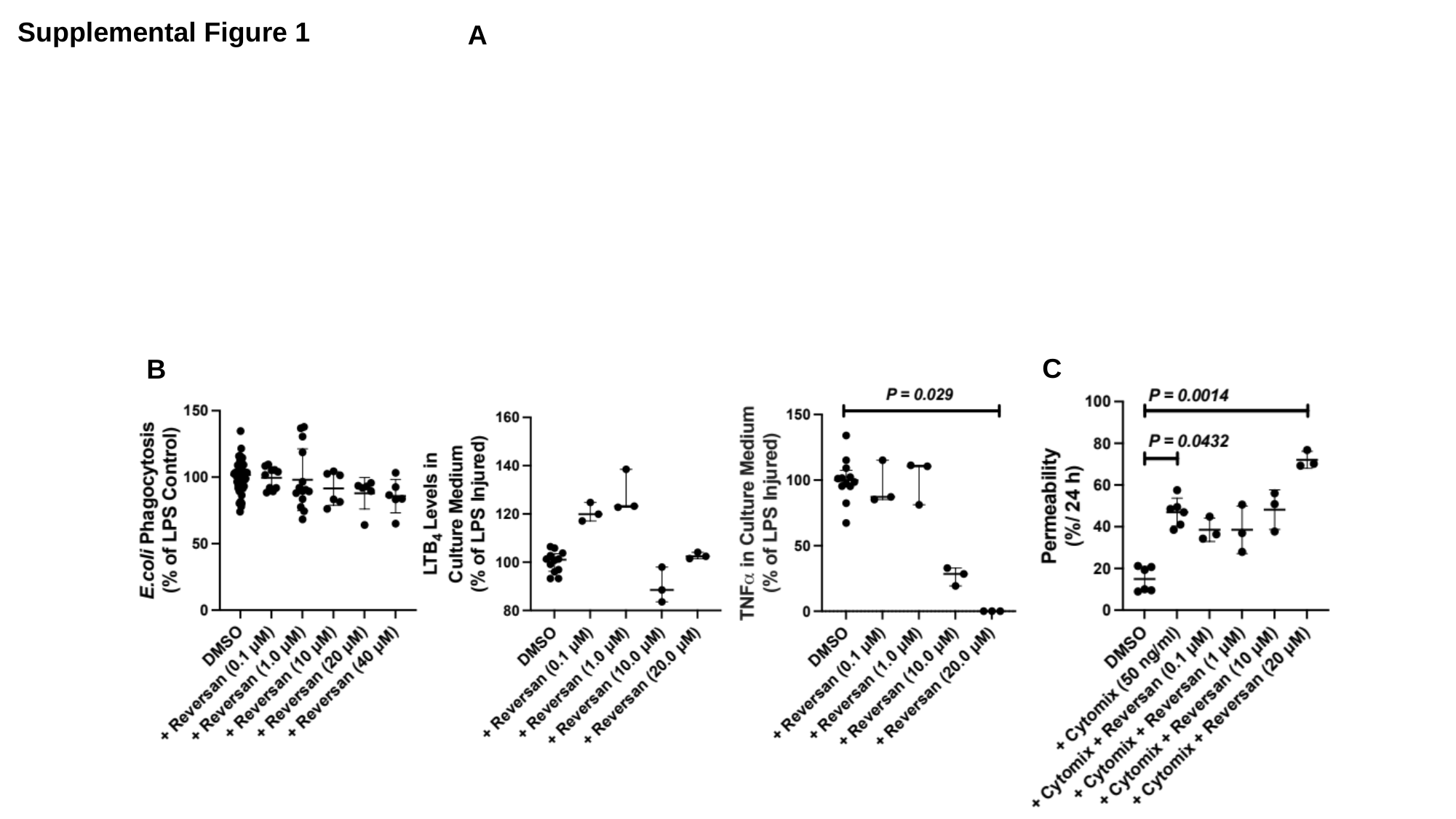

Supplemental Figure 1
A
C
B

## Slide 2
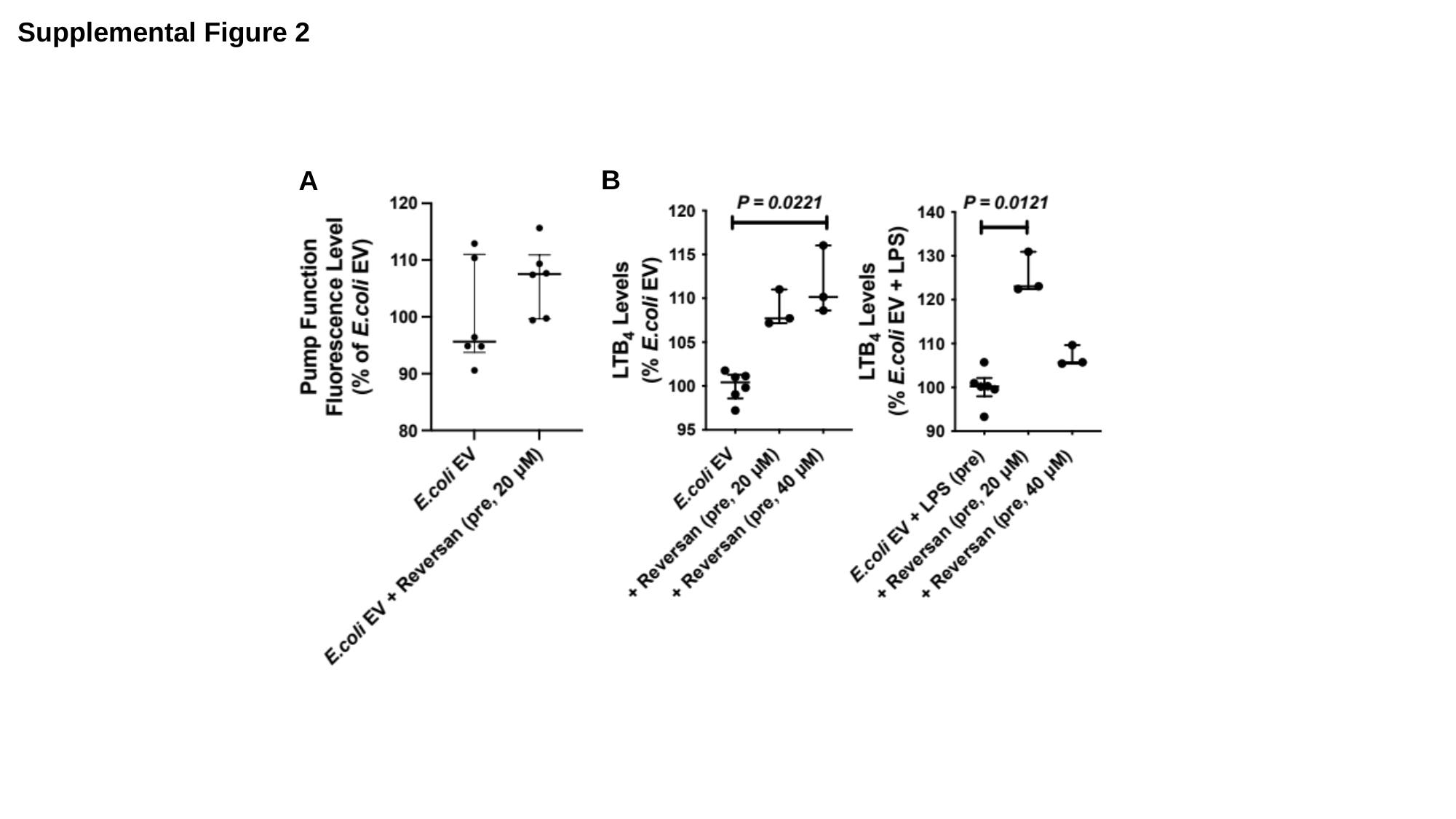

Supplemental Figure 2
B
A

## Slide 3
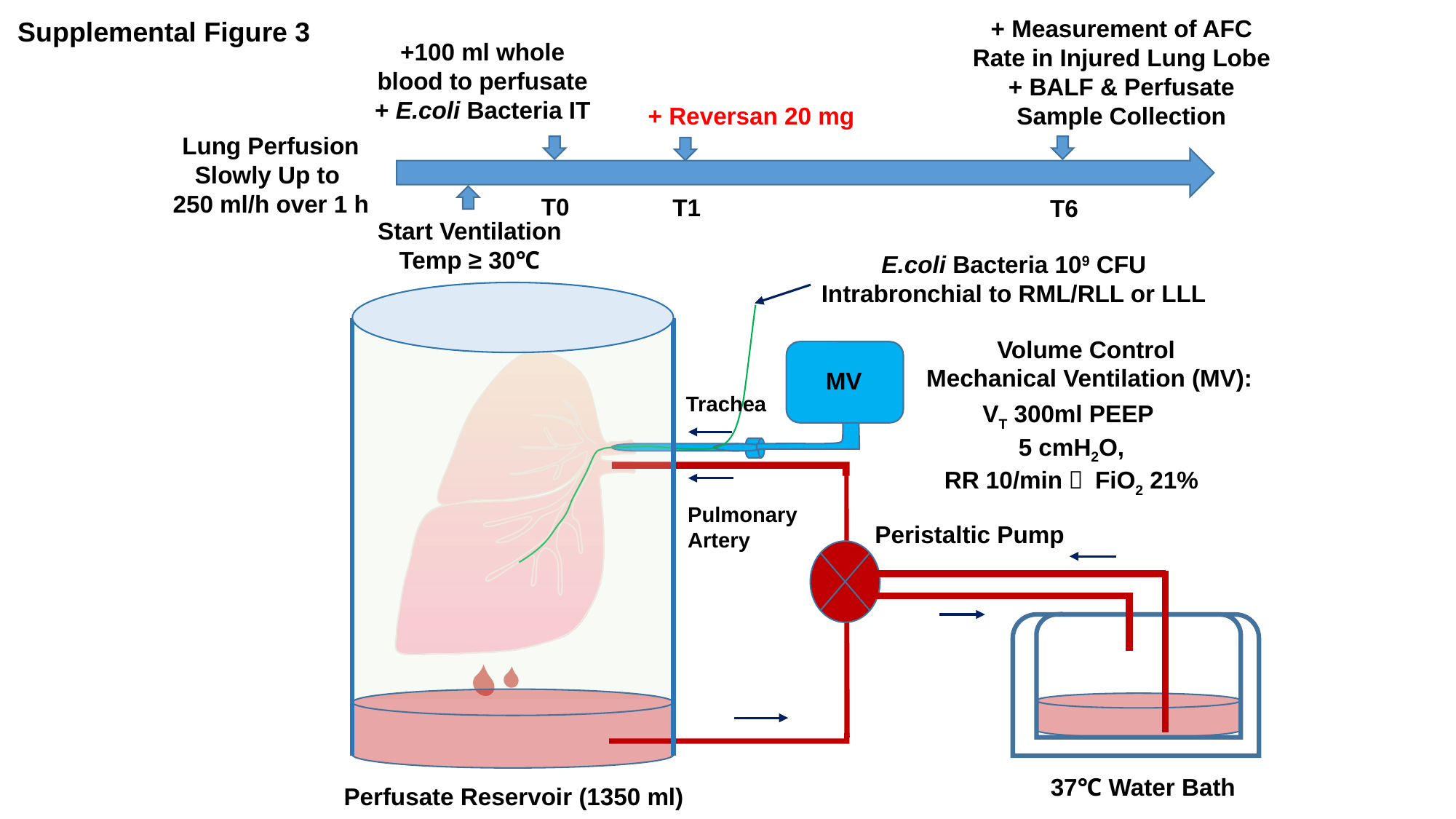

+ Measurement of AFC Rate in Injured Lung Lobe
+ BALF & Perfusate Sample Collection
+100 ml whole blood to perfusate
+ E.coli Bacteria IT
Lung Perfusion
Slowly Up to
250 ml/h over 1 h
T0
T6
Start Ventilation Temp ≥ 30℃
E.coli Bacteria 109 CFU Intrabronchial to RML/RLL or LLL
Volume Control
Mechanical Ventilation (MV):
MV
VT 300ml PEEP
5 cmH2O,
RR 10/min， FiO2 21%
Peristaltic Pump
37℃ Water Bath
Perfusate Reservoir (1350 ml)
Supplemental Figure 3
+ Reversan 20 mg
T1
Trachea
Pulmonary
Artery

## Slide 4
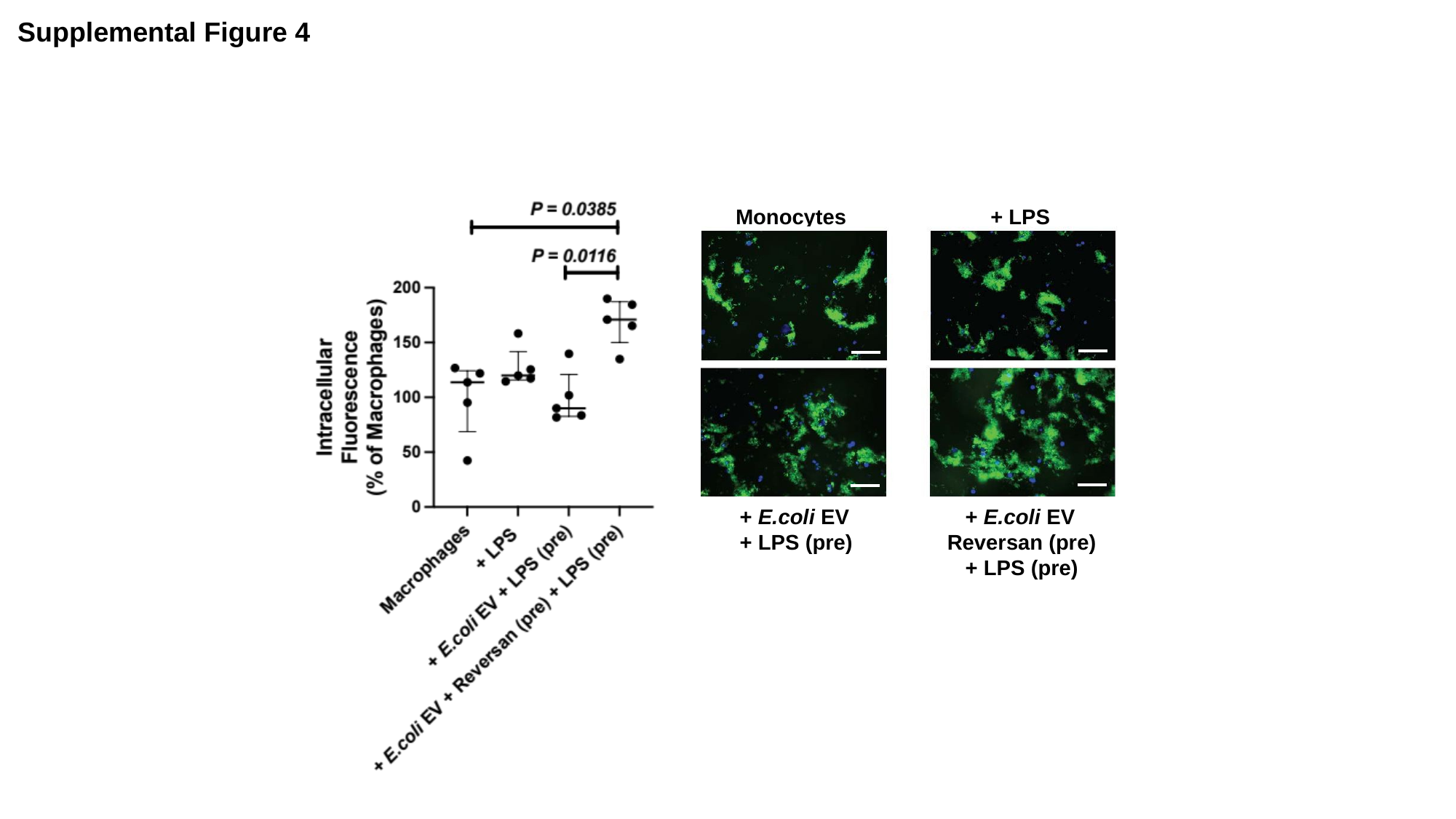

Supplemental Figure 4
 + LPS
Monocytes
 + E.coli EV
Reversan (pre)
 + LPS (pre)
+ E.coli EV
+ LPS (pre)

## Slide 5
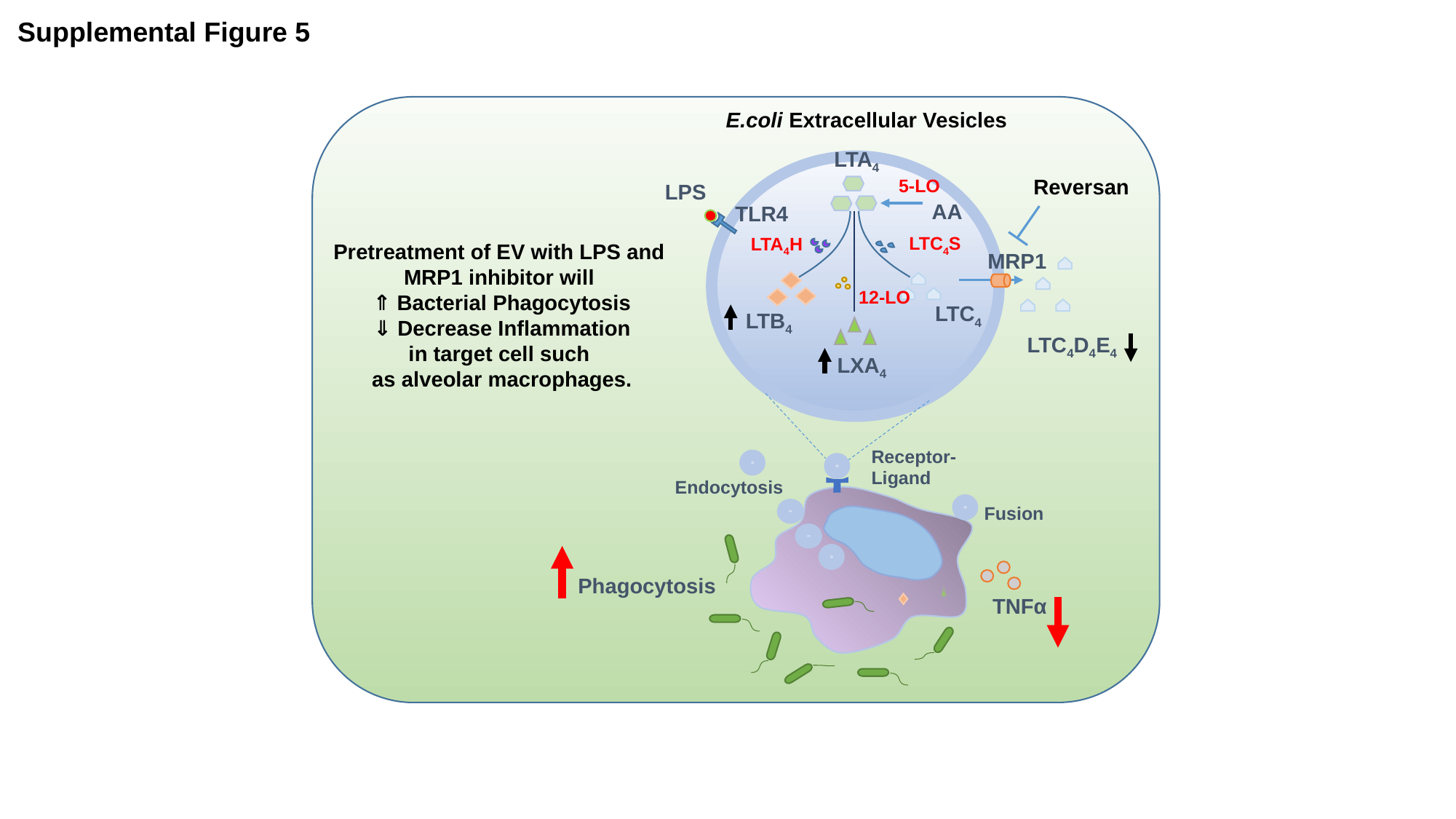

Supplemental Figure 5
E.coli Extracellular Vesicles
LTA4
MRP1
LTC4D4E4
LTC4
LTB4
LXA4
LTA4H
LTC4S
Reversan
5-LO
LPS
TLR4
AA
Pretreatment of EV with LPS and
MRP1 inhibitor will
⇑ Bacterial Phagocytosis
⇓ Decrease Inflammation
in target cell such
as alveolar macrophages.
12-LO
Receptor-Ligand
Phagocytosis
Endocytosis
Fusion
TNFα
