# Supplementary material for: Potential role of extracellular vesicles in bacterial phagocytosis during Escherichia coli pneumonia in ex vivo perfused human lungs
Source: Intensive Care Med Exp. 2026 Apr 29;14:56. doi: 10.1186/s40635-026-00902-8 (PMC13129058; doi:10.1186/s40635-026-00902-8)
Supplement: Supplementary file 2 — Additional file2 (DOCX 22 KB) [file 40635_2026_902_MOESM2_ESM.docx]

**ONLINE SUPPLEMENT**

**Potential Role of Extracellular Vesicles in Bacterial Clearance**

**During *Escherichia coli* Pneumonia In *Ex Vivo* Perfused Human Lungs**

# Hongli He, Shinji Sugita, Qi Hao, Wonjung Hwang, Li Zhou, Yoshifumi Naito, Masaru Shimizu, Sandip Mukherjee, Michael A. Matthay, Jae-Woo Lee

**LTA_4_H and LTC_4_S mRNA Levels in Extracellular Vesicles**

To determine LTA_4_H and LTC_4_S expression in nEVs and *E.coli* EVs, total RNA was reverse transcribed to cDNA using the High-Capacity RNA-to-cDNA kit (Applied Biosystems, Foster City, CA). The primers LTA_4_H ([Hs01075871_m1](https://www.thermofisher.com/taqman-gene-expression/product/Hs01075871_m1?CID=&ICID=&subtype=)), LTC_4_S ([[Hs00168529_m1](https://www.thermofisher.com/taqman-gene-expression/product/Hs00168529_m1?CID=&ICID=&subtype=)](https://www.thermofisher.com/order/genome-database/details/ceprimer/Hs00565453_CE?CID=&ICID=&subtype=) ), and GAPDH (Hs03929097_g1) were purchased from Thermo Fisher Scientific. Quantitative polymerase chain reaction (qPCR) was performed in a StepOnePlus System (Applied Biosystems) using TaqMan Fast Advanced Master Mix (Applied Biosystems). PCR activation was at 95˚C for 20 s, followed by 40 cycles of 1 s at 95˚C to denature, and 20 s at 60˚C to extend. Reactions were performed in triplicates and relative changes in target gene expression were normalized to the expression levels of GAPDH and calculated using the 2^(–△△Ct)^ method.

**Effect of LPS and MRP1 inhibitor on Leukotriene Levels in Extracellular Vesicles**

LTB_4_ and cysteinyl leukotrienes (CysLTs or LTC_4_D_4_E_4_) were measured in nEVs and *E.coli* EVs using ELISA kits (LTB_4_, R&D Systems, MN; CysLT, Cayman, MI). EVs were pretreated with LPS (*Escherichia coli* 0111:B4; Sigma–Aldrich, MO) at 10, 100, and 500 ng/ml for 24 h to determine the optimal dose required to raise LTB_4_ levels. *E.coli* EVs were then treated with a MRP1 inhibitor, Reversan, at the dose of 20 and 40 μΜ with or without LPS at 100 ng/ml for 24 h to assess LTB_4_ and Lipoxin A_4_ (LXA_4_, Cayman, MI) expression. In all experiments using Reversan, an equivalent amount of DMSO used to solubilize Reversan was added to the EVs as a carrier control. For ELISAs, we currently cannot distinguish extracellular/surface-associated LTs from intravesicular sources due to the methodology used to lysis the EVs; EV preparations were treated with RIPA lysis buffer (Thermo Fisher Scientific Pierce) supplemented with Protease Inhibitor Cocktail Set III (Calbiochem).

**Western Blot Analyses**

To confirm the effects of LPS and MRP1 inhibitor, receptors for TLR4 and MRP1 were detected in nEVs and *E.coli* EVs using Western blot analyses. nEVs and *E.coli* EVs were lysed with RIPA cell lysis buffer (Thermo Scientific) containing protease inhibitors (Sigma-Aldrich) to extract total protein. For all Westerns, 23.25 μl of the EV preparations were loaded onto the gel: for nEV (~23.9 ug of protein) and for *E.coli* EVs (~39.5 ug of protein). The Western blots were initially loaded by equal volume of the EV sample. The protein samples were heated at 70℃ for 10 min with Invitrogen NP0007 NuPAGE LDS Sample Buffer and separated out by 3 - 8% Invitrogen NuPAGE Gel under 150 voltage for 1 h. Total protein was transferred to the membrane (Invitrogen IB23001 iBlot 2 NC Regular Stacks) at 20 voltage for 7 min twice. Membrane was blocked in Starting Block buffer (Thermo Scientific, Waltham, MA, USA) for 1 h at room temperature on a shaker and then incubated with the primary mouse anti-human MRP1 antibody (1:1000 dilution, Abcam, Cambridge, MA), rabbit anti-human TLR4 antibody (1:250 dilution, Abcam, Cambridge, MA), or mouse anti-human ꞵ-actin antibody (1:40000 dilution, Sigma-Aldrich) solution overnight at 4℃ on a shaker. Membrane were washed and treated with secondary anti-rabbit IgG or anti-mouse IgG conjugated with horseradish peroxidase (1:10000 dilution, Jackson ImmunoResearch, West Grove, PA) solutions for 1 h at room temperature on a shaker. The target protein was detected using Pierce ECL Western Blotting Substrate (Thermo Scientific, Waltham, MA, USA). Images were obtained using ChemiDoc MP Imaging System (Bio-Rad, Hercules, CA, USA).

For canonical EV markers and loading controls (CD9, CD63 and βActin), Western blots were performed. Equal volume (23.25 μl of EV) were separated using SDSPAGE on NuPAGE 4-12% BisTris gel and transferred onto a PVDF membrane using the XCell II Blot module (Life Technologies). Membranes were blocked in Licor Intercept (TBS) Blocking Buffer (LI-COR Biosciences) and then probed with specific primary antibodies in Intercept® T20 (TBS) Antibody Diluent (LI-COR Biosciences), followed by fluorophore-linked secondary antibodies. Fluorescent acquisition was done on GE Amersham Molecular Dynamics Typhoon Molecular Imager. Antibodies: (1) anti-CD63 (Invitrogen), anti-Actin beta (Sigma), anti- CD9 (Invitrogen), D ἀ R- 680 Alexa Fluor® 680 AffiniPure Donkey Anti-Rabbit IgG (H+L) (Jackson Immunoresearch) and D ἀ M - IRDye® 800CW Donkey anti-Mouse IgG (Jackson Immunoresearch)

**Effect of Extracellular Vesicles on Phagocytosis of GFP-labeled *E.coli* Bacteria by Raw264.7 Cells or Human Monocytes**

For the initial experiments, 2.5 × 10^5^ Raw264.7 cells or human macrophages, collected from healthy donors, were seeded in 24-well plates and treated with nEVs or *E.coli* EVs with or without LPS at a volume of 25, 50, and 75 μl for 24 h to choose the optimal amount of EVs to use. All subsequent experiments were performed using 75 μl of EVs. After 24 h, the cells were gently washed with PBS twice, then 500 μl of antibiotics free culture medium containing 10% FBS and 2.5 × 10^6^ CFU of GFP-labeled *E.coli* bacteria (ATCC® 25922 GFPTM) were added to each well and incubated at 37℃ for 90 min. After the incubation, Raw264.7 cells or human macrophages were washed and then stained with mounting media with DAPI (Vectashield Mounting Medium with DAPI, Vector Laboratories, CA) for immunofluorescence. Intracellular *E.coli* bacteria levels were evaluated using fluorescence microscopy; intracellular fluorescence intensity was measured with a FLUOstar OPTIMA fluorescent plate reader following lysis of the cells. For these subsequent experiments, EVs were incubated with LPS (100 ng/m), Reversan (20 μΜ), or Zileuton (10 μΜ, TOCRIS Bioscience, Minneapolis, MD), a 5-LO inhibitor, for 24 h. The pretreated EVs were then cocultured with Raw264.7 cells or human macrophages for 24 h to evaluate phagocytosis activity. After 24 h, the medium was collected to measure TNFα, LTB_4_ and LXA_4_ levels by ELISA. To prevent the synthesis of LTB_4_ and LXA_4_ in *E.coli* EVs after pretreatment with LPS with or without Reversan, the 5-LO inhibitor Zileuton (TOCRIS Bioscience, Minneapolis, MN) were incubated with *E.coli* EVs for 24 h at a dose of 5, 10, 20 μM to determine the optimal dose for inhibition of LTB_4_ synthesis prior to experiments with Raw264.7 cells or human macrophages. Note: Zileuton also inhibit cysteinyl LTs or LTC_4_D_4_E_4_ synthesis which may affect the interpretation on inflammation such as TNFα levels.

In EV pretreatment experiments, 75 μl of *E.coli* were incubated with LPS with or without Reversan in a final volume of 77 μl giving a final conc. of 100 ng/ml of LPS and 20 μM Reversan. In co-incubation experiments with Raw264.7 cells, the 77 μl of *E.coli* EVs were added to 250,000 Raw264.7 cells in a volume of 750 μl for 24 h. The final concentration of the LPS and/or Reversan in the co-incubation experiments were 9 ng/ml and/or 2 μM respectively. As controls, the diluted concentration of LPS or Reversan had no effect on GFP labelled *E.coli* bacterial phagocytosis when given alone to Raw264.7 cells. However, the DMSO used to solubilized Reversan had a small but statistically significant effect on EV LTB_4_ and LXA_4_ levels despite a volume% of <0.3% for EV pretreatment experiments and a volume% <0.03% for co-incubation experiments with Raw264.7 cells. In experiments using Reversan, the same volume% of DMSO was given to the control cells. We did not attempt to remove LPS, Reversan or DMSO following pretreatment of the EVs; we previously found that further washing and re-isolating the EVs following exposure to LPS, Reversan or DMSO would lead to EV yield loss of >50%.

**MRP1 Pump Assay**

To confirm the effect of LPS and/or MRP1 inhibitor on MRP1 pump function, a fluoroimetric MDR assay kit (Abcam, Cambridge, UK) was used. P-glycoprotein (Pgp, MDR1) and MRP1, members of the ABC transporter family, are cell membrane proteins and function as ATP dependent drug efflux pumps. This MDR assay kit uses a fluorescent MDR indicator dye to assay these two pump activities. The hydrophobic fluorescent dye rapidly penetrates cell membranes and becomes trapped in the EVs. In the MDR1 and/or MRP1-expressing EVs, this dye is extruded by MDR transporters, thus decreasing vesicular fluorescence intensity. However, when MDR1 and/or MRP1 pump-activity are suppressed, their intracellular dye cannot be pumped out, thus increasing intra-vesicular fluorescence intensity.
